# Infiltration and Nerve Block in Painful Shoulder: Current Perspectives and Trends

**DOI:** 10.1055/s-0044-1792098

**Published:** 2025-04-11

**Authors:** Paulo Henrique Schmidt Lara, Guilherme Martinez, Fabrício Infanti, Alberto de Castro Pochini, Benno Ejnisman, Paulo Santoro Belangero

**Affiliations:** 1 Centro de Traumatologia do Esporte (CETE), Departamento de Ortopedia e Traumatologia, Escola Paulista de Medicina, Universidade Federal de São Paulo (DOT-EPM/UNIFESP), São Paulo, SP, Brasil; 2 Departamento de Ortopedia e Traumatologia, Escola Paulista de Medicina, Universidade Federal de São Paulo (DOT-EPM/UNIFESP), São Paulo, SP, Brasil

**Keywords:** athrosis, infiltration, nerve block, shoulder, tendinopathy

## Abstract

**Objective** To investigate the use of infiltration and nerve block in shoulder pain treatment by shoulder surgery specialists and compared the results with previous research conducted by our group in 2017.

**Methods** The present study consisted of a cross-sectional analysis of shoulder surgery specialists to investigate the use of infiltration and nerve block in treating shoulder pain. The survey employed a structured questionnaire addressing the clinical practice regarding these procedures. We collected and analyzed the data using descriptive statistics and associated analyses between variables, such as patient age and type of procedure performed.

**Results** The results revealed a high rate of infiltrations and nerve blocks for shoulder pain treatment, especially in patients over 40. The use of steroids and hyaluronic acid in infiltrations was common, particularly in shoulder osteoarthritis and partial rotator cuff tears. However, ultrasound guidance during the procedures was not frequent. Two thirds of respondents performed nerve blocks, mainly of the suprascapular nerve, with a low complication rate.

**Conclusion** The present study highlighted the prevalence and trends in clinical practice regarding infiltrations and nerve blocks in shoulder pain treatment. Despite the gaps identified, such as the low use of ultrasound guidance, the results provide valuable insights to improve therapeutic approaches and to consider the adoption of imaging technologies in the field.

## Introduction

Shoulder pain complaints frequently affect adult patients, with a prevalence ranging from 7 to 34%, with the population over 40 being at the greatest risk. 1 2 It is estimated that 10 to 16% of the population will have more than one episode of painful shoulder throughout their lives. 1 3 The medical practice questions the treatment, monitoring, and recovery of this type of complaint, and factors such as age between 45 and 54 and pain lasting more than 3 months indicate a poor prognosis. 1 4 Refractoriness and recurrence of the complaint after 1 year are complications present in approximately 40 to 50% of patients. 4 When studying the pathophysiology of conditions related to painful shoulder, degenerative osteotendinous diseases and inflammatory changes, including tendonitis, with or without calcification, are essential. 5 It is worth noting that the degeneration stage and comorbidity severity are directly associated with the prognosis and outcome. 5 6

Infiltration and nerve block are procedures with proven effectiveness in recent years for controlling shoulder pain symptoms, in addition to the several drugs that are part of the repertoire of physicians managing these complaints. 7 8 9 Infiltration sites, imaging guidance, and indications also vary. 9 10 The objective of the present study was to evaluate how shoulder specialists have used infiltration and nerve block in their daily practice.

## Materials and Methods

This cross-sectional study, characterized as a single-time, intersectional survey research, adopted a non-probabilistic convenience sampling. The Ethics Committee of our institution approved the study under number 71476223.3.0000.5505.

We administered a questionnaire previously developed by the authors ( Supplementary Appendix 1 ) during the 8th Closed Meeting of the Brazilian Society of Shoulder and Elbow Surgery, held from August 17 to 19, 2023.

One hundred thirty-four orthopedists specialized in shoulder and elbow surgery answered the questionnaire out of a total of 300 registered participants, resulting in a response rate of 44%. To preserve confidentiality, we did not identify the orthopedists and asked them to answer the questionnaire only once. We distributed the questionnaires during the breaks of the three days of the congress and allowed responses virtually (through Google Forms) and in person.

We described the characteristics assessed in the questionnaire as absolute and relative frequencies; then, we analyzed the procedures of interest regarding the professionals' experience using likelihood-ratio tests. Finally, we related the use of steroids in different types of procedures with the administration of hyaluronic acid during infiltrations and assessed the association using Fisher exact tests.

Each survey had questions about the professionals' experience and the number of infiltrations performed, and we evaluated their association through Chi-squared tests, as recommended by Kirkwood and Sterne (2006).

Data statistical analysis employed the IBM SPSS Statistics for Windows (IBM Corp., Armonk, NY, USA) software, version 22.0. We performed data tabulation in the Microsoft Excel 2013 (Microsoft Corp., Redmond, WA, USA) software. All tests considered a significance level of 5%.

## Results

Among the respondents, 29.9% had over 10 years of experience in shoulder surgery; 20.1% had between 5 and 10 years; 37.3% had from 1 to 5 years; and 12.7% had less than 1 year of experience. In the study population, 48.5% reported 10 to 30 infiltration indications within the previous year.

Indications for subacromial and glenohumeral steroid infiltration accounted for 92.5% and 80.6% of the total infiltration indications, respectively. Regarding subacromial infiltration, 50.7% opted for lateral approaches, 26.9% for posterior approaches, and 3% for anterior approaches; 18.4% participants mentioned other approaches. For glenohumeral infiltration, 53.7% used anterior approaches, 29.1% used posterior approaches, and 17.2% used different approaches.

Most respondents (73.1%) did not use ultrasound guidance for infiltration, and 75.4% performed subacromial infiltration in the office. As for glenohumeral infiltration, 45.5% performed them in the office, while 19.4% did it in the surgical center.

Most respondents (86.6%) used hyaluronic acid for infiltrations, including 22% for treating shoulder osteoarthritis alone and 18.7% for treating partial rotator cuff tears/tendinopathies. The main complication reported by respondents was postinfiltration pain, accounting for 50% of the complications in the subacromial region and 44.8% in the articular region.

Regarding shoulder nerve block, 66.4% of respondents performed the procedure while 33.6% did not; 44.8% blocked the suprascapular nerve, and 29.9% added an axillary nerve block. Among the participants performing blocks, only 32.1% used ultrasound for guidance. Most respondents reported no post-procedure complications (36.6%). The most reported complication was local pain, corresponding to 24.6% of cases.

Table 1 shows that the higher number of infiltrations in the last year is associated with the professionals' greater experience ( *p* < 0.001); professionals with less experience used fewer steroids in subacromial infiltrations and performed fewer infiltrations with hyaluronic acid ( *p* = 0.004 and *p* = 0.041 respectively), while orthopedists with greater experience performed statistically less nerve blocks ( *p* = 0.001).

**Table 1:** Description of the professional conduct per experience and results of association tests

Variable	1) How many years of experience do you have in shoulder surgery?				Total	*p*
	< 1 year	1–5 years	5–10 years	> 10 years		
**2) How many infiltrations did you perform in the last 12 months?**						**< 0.001**
None	4 (23.5)	1 (2)	0 (0)	0 (0)	5 (3.7)	
1–10	9 (52.9)	27 (54)	0 (0)	4 (10)	40 (29.9)	
10–30	3 (17.6)	18 (36)	22 (81.5)	22 (55)	65 (48.5)	
> 40	1 (5.9)	4 (8)	5 (18.5)	14 (35)	24 (17.9)	
**8) Do you perform ultrasound-guided infiltration?**						0.079
No	9 (52.9)	42 (84)	19 (70.4)	28 (70)	98 (73.1)	
Yes	8 (47.1)	8 (16)	8 (29.6)	12 (30)	36 (26.9)	
**12) Which medication(s) do you use for subacromial infiltration?**						**0.004**
Without steroid	5 (29.4)	3 (6)	2 (7.4)	0 (0)	10 (7.5)	
With steroid	12 (70.6)	47 (94)	25 (92.6)	40 (100)	124 (92.5)	
**13) Which medication(s) do you use for glenohumeral infiltration?**						0.169
Without steroid	4 (23.5)	14 (28)	3 (11.1)	5 (12.5)	26 (19.4)	
With steroid	13 (76.5)	36 (72)	24 (88.9)	35 (87.5)	108 (80.6)	
**14) Which medication(s) do you use for acromioclavicular infiltration?**						0.542
Without steroid	2 (12.5)	2 (4)	1 (3.7)	1 (2.5)	6 (4.5)	
With steroid	14 (87.5)	48 (96)	26 (96.3)	39 (97.5)	127 (95.5)	
**16) Do you perform hyaluronic acid infiltration?**						**0.041**
No	6 (35.3)	6 (12)	1 (3.7)	5 (12.5)	18 (13.4)	
Yes	11 (64.7)	44 (88)	26 (96.3)	35 (87.5)	116 (86.6)	
**20) Do you perform nerve block?**						**0.001**
No	4 (23.5)	16 (32)	3 (11.1)	22 (55)	45 (33.6)	
Yes	13 (76.5)	34 (68)	24 (88.9)	18 (45)	89 (66.4)	
**26) Do you perform ultrasound-guided nerve block?**						0.666
No	10 (62.5)	32 (65.3)	16 (64)	11 (50)	69 (61.6)	
Yes	6 (37.5)	17 (34.7)	9 (36)	11 (50)	43 (38.4)	

**Note:** Likelihood-ratio test.

Table 2 reveals no statistically significant association between steroid use in these procedures and the performance of hyaluronic acid infiltration ( *p* > 0.05).

**Table 2:** Description of the performance of hyaluronic acid infiltration according to steroid use in each type of procedure and results of the association tests

Variable	16) Do you perform hyaluronic acid infiltration?		Total	*p*
	No	Yes		
**12) Which medication(s) do you use for subacromial infiltration?**				0.133
Without steroid	3 (16.7)	7 (6)	10 (7.5)	
With steroid	15 (83.3)	109 (94)	124 (92.5)	
**13) Which medication(s) do you use for glenohumeral infiltration?**				> 0.999
Without steroid	3 (16.7)	23 (19.8)	26 (19.4)	
With steroid	15 (83.3)	93 (80.2)	108 (80.6)	
**14) Which medication(s) do you use for acromioclavicular infiltration?**				0.590
Without steroid	1 (5.6)	5 (4.3)	6 (4.5)	
With steroid	17 (94.4)	110 (95.7)	127 (95.5)	

**Note:** Fisher exact test.

Table 3 reveals that respondents had statistically less experience than in the previous study ( *p* = 0.001) and the number of infiltrations performed in the last 12 months by the professionals from the current survey was statistically higher than in the previous study ( *p* < 0.001).

**Table 3:** Description of the experience and number of infiltrations in the last 12 months in the two surveys and results of the association tests

Variable	Previous study	Current study	*p*
**1) How many years of experience do you have in shoulder surgery?**			**0.001**
< 1 year	17 (9.5)	17 (12.7)	
1–5 years	35 (19.6)	50 (37.3)	
5–10 years	43 (24)	27 (20.1)	
> 10 years	84 (46.9)	40 (29.9)	
**2) How many infiltrations did you perform in the last 12 months?**			**< 0.001**
≤ 10	113 (63.1)	45 (33.6)	
> 10	66 (36.9)	89 (66.4)	

**Note:** Chi-squared test.

## Discussion

The present study investigated the approach of shoulder surgery specialists in treating shoulder pain complaints, a prevalent condition with a significant impact on patients' quality of life. 11 12 The research aimed to analyze how specialized orthopedists have used infiltration and nerve block in their daily practice, offering a comprehensive view of trends and practices in the current medical community and comparing these results from 2020 up to now to those from the previous study by our group, which included a similar questionnaire.

The results revealed a diverse sample of orthopedists, covering different experience levels, which is crucial to understanding the variations in clinical practices. The predominance of patients over 40, as identified in the study, was consistent with the literature, highlighting the relevance of this condition in an older population. 13 14

The study highlighted the prevalence of steroid use in subacromial and glenohumeral infiltrations, indicating a practice in line with the current literature. 15 Preferences regarding the lateral approach in subacromial infiltrations and anterior approach points in glenohumeral infiltrations reflected the diversity in techniques used by specialists. The incorporation of hyaluronic acid in infiltrations was notable, with a significant percentage of professionals adopting this approach, mainly for treating shoulder osteoarthritis and partial rotator cuff tears/tendinopathies. 16 17 This trend suggested a search for more comprehensive and conservative therapies in shoulder pain treatment.

Most respondents did not use ultrasound as a guide during the infiltration procedure, revealing a possible gap in incorporating imaging technologies in clinical practice. This finding raises discussions about the efficacy and need for imaging methods to improve procedural accuracy. 18 19 However, there was a significant 15% increase in the use of ultrasound as an auxiliary examination in shoulder infiltrations compared to the previous study carried out in 2020 by our group.

Shoulder nerve block is a common practice among specialists, and the suprascapular nerve is the most frequently blocked. 20 The preference for not using ultrasound to guide the procedure may indicate confidence in the conventional technique or the need for greater awareness of the benefits of ultrasound in this context. 21 The reported complication rate is relatively low, and local pain was the most common complication. This result suggests a safe approach to these procedures, but a detailed analysis of complications may provide valuable insights to improve techniques and reduce risks. 22 23

The association between time of experience and the number of infiltrations performed is intriguing. This study had more specialists with a shorter time since training completion compared with the previous research. More experienced professionals statistically performed fewer nerve blocks, indicating a potential preference for other therapeutic options or greater effectiveness in treatment decisions. It is worth noting that the 44% response rate may introduce a selection bias in the sample, limiting the generalizability of the results. Furthermore, the conventional approach during conferences may have influenced participants' responses since they may have been more likely to discuss traditional practices.

We observed similarities and advances when comparing this study with previous research from 2017 during the 5 ^th^ Closed Meeting of the Brazilian Shoulder and Elbow Society, in which 179 orthopedic shoulder and elbow specialists answered the questionnaire. 24

These two studies highlighted the widespread adoption of infiltration and nerve block as effective therapies to mitigate shoulder pain symptoms. However, the present study expanded on this analysis by addressing the relationship between orthopedists' experience and their clinical practices. In addition, both studies highlighted the underutilization of ultrasound as an adjunct tool in infiltration procedures, indicating a potential failure to integrate imaging technologies into clinical routine.

The two studies concluded that both infiltration and nerve block of the shoulder are safe procedures, with a low rate of reported complications. However, this most recent study added a new perspective by exploring the incorporation of hyaluronic acid in infiltrations, suggesting a trend toward more comprehensive and conservative therapies. In addition, it adds prolotherapy and bone marrow aspirate (BMA) concentrate as treatment options, representing innovative therapies still under evaluation for their true relevance and efficacy in shoulder pain treatment.

## Conclusion

The prevalence of steroid and hyaluronic acid infiltrations indicated a trend toward innovative and conservative approaches. The underutilization of ultrasound highlighted a potential gap in adopting imaging technologies, raising the need to evaluate the efficacy of these methods. Nerve blocks in the shoulder, especially the suprascapular nerve, were common and safe, with a low incidence of complications. The association between experience and the infiltration number suggested an evolution throughout the career, with more experienced professionals opting for fewer nerve blocks. Despite limitations, such as the 44% response rate, the study provided valuable insights to improve clinical practices in the management of shoulder pain by orthopedists.

## Supplementary Material

Supplementary Anexo 1 Supplementary Anexo 1
