# Supplementary material for: Infiltration and Nerve Block in Painful Shoulder: Current Perspectives and Trends
Source: Rev Bras Ortop (Sao Paulo). 2025 Apr 11;60(1):s00441792098. doi: 10.1055/s-0044-1792098 (PMC12020545; doi:10.1055/s-0044-1792098)
Supplement: Supplementary file 1 — Supplementary Anexo 1 [file 10-1055-s-0044-1792098-s2400151pt.pdf]

Supplementary Anexo 1

Questionário para realização do estudo “Infiltração e bloqueio de nervo no ombro doloroso: perspectivas e tendências atuais” a ser aplicado no 8° Closed Meeting da Sociedade Brasileira de Cirurgia do Ombro e Cotovelo que ocorrerá em agosto de 2023.

Trata-se de um estudo que avaliará como os ortopedistas especializados em ombro utilizam a infiltração/bloqueio de nervo na sua prática diária para o ombro doloroso.

1. Quantos anos de experiência possui na cirurgia de ombro?
   ( ) Menos de 1 ano ( ) 1-5 anos ( ) 5-10 anos ( ) Mais de 10 anos
2. Quantas infiltrações realizou nos últimos 12 meses?
   ( ) Nenhuma ( ) 1 a 10 ( ) 10 a 30 ( ) > 40
3. Após quanto tempo realiza nova infiltração?
   ( ) 1 mês ( ) 2 meses ( ) 3 meses ( ) 4 meses ou mais
4. Para qual ou quais patologias você utiliza a infiltração subacromial?
   ( ) Lesão total do manguito rotador ( ) Lesão parcial do manguito rotador - face bursal ( ) Lesão parcial do manguito rotador - face articular ( ) Tendinopatia do manguito rotador ( ) Bursite ( ) Capsulite adesiva ( ) Tendinite calcária ( ) Outras. Quais?
5. Para qual ou quais patologias você utiliza a infiltração na articulação glenoumeral?
   ( ) Lesão total do manguito rotador ( ) Lesão parcial do manguito rotador - face bursal ( ) Lesão parcial do manguito rotador - face articular ( ) Tendinopatia do manguito rotador ( ) Artrose do ombro ( ) Bursite ( ) Capsulite adesiva ( ) Sinovite ( ) Outras. Quais?
6. Em qual ponto realiza a infiltração subacromial?
   ( ) Anterior ( ) Posterior ( ) Lateral ( ) Outro. Qual?
7. Em qual ponto realiza a infiltração glenoumeral?
   ( ) Anterior ( ) Posterior ( ) Outro. Qual?
8. Realiza a infiltração guiada por ultrassonografia?
   ( ) Sim (infiltração subacromial e glenoumeral) ( ) Sim (infiltração subacromial)
   ( ) Sim (infiltração glenoumeral) ( ) Não
9. Onde você costuma realizar a infiltração subacromial?
   ( ) Consultório ( ) Centro cirúrgico
10. Onde você costuma realizar a infiltração glenoumeral?
    ( ) Consultório ( ) Centro cirúrgico
11. Qual medicamento você utiliza na infiltração subacromial?
    ( ) Corticóide ( ) Anti-inflamatório ( ) Anestésico
    ( ) PRP, proloterapia, BMA ou outras terapias regenerativas ( ) Ácido hialurônico ( ) Corticóide + Anestésico
    ( ) Outras terapias combinadas. Qual/quais?
12. Qual medicamento você utiliza na infiltração glenoumeral?
    ( ) Corticóide ( ) Anti-inflamatório ( ) Anestésico
    ( ) PRP, proloterapia, BMA ou outras terapias regenerativas ( ) Ácido hialurônico ( ) Corticóide + Anestésico
    ( ) Outras terapias combinadas. Qual/quais?
13. Se utiliza corticóide, qual a sua preferência?
    ( ) Triancinolona ( ) Dexametasona ( ) Metilprednisolona
    ( ) Hidrocortisona ( ) Betametasona ( ) Outro. Qual/quais?
14. Realiza infiltração de ácido hialurônico?
    ( ) Não ( ) Sim
15. Se realiza infiltração de ácido hialurônico, para quais doenças o utiliza?
    ( ) Lesão total do manguito rotador ( ) Lesão parcial do manguito rotador
    ( ) Tendinopatias ( ) Artrose do ombro ( ) Capsulite adesiva ( ) Tendinite calcária ( ) Outras. Qual/quais?
16. Quais as principais complicações na sua prática de infiltração subacromial?
    ( ) Infecção ( ) Dor após infiltração ( ) Despigmentação cutânea
    ( ) Calcificação pericapsular ( ) Lesão neurovascular ( ) Outras. Qual/quais?
    ( ) Nenhuma
17. Quais as principais complicações na sua prática de infiltração articular ?
    ( ) Infecção ( ) Dor após infiltração ( ) Despigmentação cutânea
    ( ) Calcificação pericapsular ( ) Lesão neurovascular ( ) Outras. Qual/quais?
    ( ) Nenhuma
18. Realiza bloqueio de nervo do ombro?
    ( ) Não ( ) Sim
19. Se realiza bloqueio de nervo, qual nervo do ombro costuma bloquear?
    ( ) N. supraescapular ( ) N. axilar ( ) N. supraescapular + N. axilar ( ) Nenhum
20. Quais medicamentos utiliza no bloqueio do nervo do ombro?
    ( ) Corticóide ( ) Anestésico ( ) Corticóide + anestésico ( ) Outros. Qual/quais?
21. Se realiza bloqueio de nervo axilar , para quais patologias o utiliza?
    ( ) Lesão total do manguito rotador ( ) Lesão parcial do manguito rotador
    ( ) Tendinopatia do manguito rotador ( ) Artrose do ombro ( ) Capsulite adesiva ( ) Tendinite calcária ( ) Outras. Qual/quais?
22. Se realiza bloqueio de nervo supraescapular , para quais patologias o utiliza?
    ( ) Lesão total do manguito rotador ( ) Lesão parcial do manguito rotador

 © 2025. The Author(s).

( ) Tendinopatia do manguito rotador ( ) Artrose do ombro ( ) Capsulite adesiva ( ) Tendinite calcária ( ) Outras. Qual/quais?

23. Se realiza bloqueio de nervo combinado (Axilar + Supraescapular) , para quais patologias o utiliza?
( ) Lesão total do manguito rotador ( ) Lesão parcial do manguito rotador
( ) Tendinopatia do manguito rotador ( ) Artrose do ombro ( ) Capsulite adesiva ( ) Tendinite calcária ( ) Outras. Qual/quais?

24. Realiza bloqueio de nervo do ombro guiado por ultrassonografia?
( ) Não ( ) Sim

25. Quais as principais complicações na sua prática de bloqueio?
( ) Infecção ( ) Dor após bloqueio ( ) Despigmentação cutânea
( ) Lesão neurovascular ( ) Outras. Qual/quais? ( ) Nenhuma

 © 2025. The Author(s).
