# Supplementary material for: Infiltration and Nerve Block in Painful Shoulder: Current Perspectives and Trends
Source: Rev Bras Ortop (Sao Paulo). 2025 Apr 11;60(1):s00441792098. doi: 10.1055/s-0044-1792098 (PMC12020545; doi:10.1055/s-0044-1792098)
Supplement: Supplementary file 2 — Supplementary Appendix 1 [file 10-1055-s-0044-1792098-s2400151en.pdf]

Supplementary Appendix 1

Questionnaire for the study "Infiltration and nerve block in the painful shoulder: perspectives and current trends" applied at the VIII Closed Meeting of the Brazilian Society of Shoulder and Elbow Surgery, in August 2023.

This study will evaluate how orthopedists specializing in shoulder use infiltration/nerve block in their daily practice for painful shoulders.

1. How many years of experience do you have in shoulder surgery?
( ) Less than 1 year ( ) 1 to 5 years ( ) 5 to 10 years ( ) Over 10 years
2. How many infiltrations did you perform in the last 12 months?
( ) None ( ) 1 to 10 ( ) 10 to 30 ( ) >40
3. After how long do you perform a new infiltration?
( ) 1 month ( ) 2 months ( ) 3 months ( ) 4 months or more
4. For which condition(s) do you perform subacromial infiltration?
( ) Complete rotator cuff tear ( ) Partial rotator cuff tear - bursal face ( ) Partial rotator cuff tear - articular face ( ) Rotator cuff tendinopathy ( ) Bursitis ( ) Adhesive capsulitis ( ) Calcific tendonitis ( ) Other. Which ones?
5. For which condition(s) do you perform glenohumeral infiltration?
( ) Complete rotator cuff tear ( ) Partial rotator cuff tear - bursal face ( ) Partial rotator cuff tear - articular face ( ) Rotator cuff tendinopathy ( ) Shoulder arthrosis ( ) Bursitis ( ) Adhesive capsulitis ( ) Synovitis ( ) Other. Which ones?
6. In which point do you perform subacromial infiltration?
( ) Anterior ( ) Posterior ( ) Lateral ( ) Other. Which one?
7. In which point do you perform glenohumeral infiltration?
( ) Anterior ( ) Posterior ( ) Other. Which one?
8. Do you perform ultrasound-guided infiltration?
( ) Yes (subacromial infiltration and glenohumeral) ( ) Yes (subacromial infiltration)
( ) Yes (glenohumeral infiltration) ( ) No
9. Subacromial infiltration?
( ) Medical office ( ) Surgical center
10. Where do you usually perform glenohumeral infiltration?
( ) Medical office ( ) Surgical center
11. Which medication(s) do you use for subacromial infiltration?
( ) Steroid ( ) Anti-inflammatory ( ) Anesthetic
( ) PRP, prolotherapy, BMA , or other regenerative techniques ( ) Hyaluronic acid ( ) Steroid + Anesthetic
( ) Other combined therapies. Which one(s)?
12. Which medication(s) do you use for glenohumeral infiltration?
( ) Steroid ( ) Anti-inflammatory ( ) Anesthetic
( ) PRP, prolotherapy, BMA, or other regenerative techniques ( ) Hyaluronic acid ( ) Steroid + Anesthetic
( ) Other combined therapies. Which one(s)?
13. If you use steroids, which one do you prefer?
( ) Triamcinolone ( ) Dexamethasone ( ) Methylprednisolone ( ) Hydrocortisone ( ) Betamethasone ( ) Other. Which one(s)?
14. Do you perform hyaluronic acid infiltration?
( ) No ( ) Yes
15. If you perform hyaluronic acid infiltrations, for which conditions do you use it?
( ) Complete rotator cuff tear ( ) Partial rotator cuff tear
( ) Tendinopathies ( ) Shoulder arthrosis ( ) Adhesive capsulitis ( ) Calcific tendonitis ( ) Other. Which one(s)?
16. Which are the most frequent complications in your experience with subacromial infiltration?
( ) Infection ( ) Post-infiltration pain ( ) Skin depigmentation
( ) Pericapsular calcification ( ) Neurovascular injury ( ) Other. Which one(s)?
( ) None
17. Which are the most frequent complications in your experience with joint infiltration?
( ) Infection ( ) Post-infiltration pain ( ) Skin depigmentation
( ) Pericapsular calcification ( ) Neurovascular injury ( ) Other. Which one(s)?
( ) None
18. Do you perform nerve block?
( ) No ( ) Yes
19. If you perform nerve block, which shoulder nerve do you usually block?
( ) Suprascapular n. ( ) Axillary n. ( ) Suprascapular n.+ Axillary n. ( ) None
20. Which medications do you use for shoulder nerve block?
( ) Steroid ( ) Anesthetic ( ) Steroid + Anesthetic ( ) Other. Which one(s)?
21. If you perform axillary nerve block, for which conditions do you do it?
( ) Complete rotator cuff tear ( ) Partial rotator cuff tear
( ) Rotator cuff tendinopathy ( ) Shoulder arthrosis ( ) Adhesive capsulitis ( ) Calcific tendonitis ( ) Other. Which one(s)?
22. If you perform suprascapular nerve block, for which conditions do you do it?
( ) Complete rotator cuff tear ( ) Partial rotator cuff tear
( ) Rotator cuff tendinopathy ( ) Shoulder arthrosis ( ) Adhesive capsulitis ( ) Calcific tendonitis ( ) Other. Which one(s)?

 © 2025. The Author(s).

23. If you perform a combined nerve block (Axillary + Suprascapular), for which conditions do you do it?
( ) Complete rotator cuff tear ( ) Partial rotator cuff tear ( ) Rotator cuff tendinopathy ( ) Shoulder arthrosis ( ) Adhesive capsulitis ( ) Calcific tendonitis ( ) Other. Which one(s)?
24. Do you perform ultrasound-guided shoulder nerve block?
( ) No ( ) Yes
25. Which are the most frequent complications in your experience with blocks?
( ) Infection ( ) Post-block pain ( ) Skin depigmentation ( ) Neurovascular injury ( ) Other. Which one(s)? ( ) None

BMA, Bone marrow aspiration concentrate; PRP, platelet-rich plasma.

 © 2025. The Author(s).
