# Supplementary material for: Infiltration and Nerve Block in Painful Shoulder: Current Perspectives and Trends
Source: Rev Bras Ortop (Sao Paulo). 2025 Apr 11;60(1):s00441792098. doi: 10.1055/s-0044-1792098 (PMC12020545; doi:10.1055/s-0044-1792098)
Supplement: Related texts

# Research article: Infiltração e bloqueio de nervo no ombro doloroso: Perspectivas e tendências atuais

**Keywords:** artrose, bloqueio nervoso, infiltração, ombro, tendinopatia

## Abstract

**Objetivo** Investigar o uso de infiltração e bloqueio de nervo no tratamento da dor no ombro por especialistas em cirurgia de ombro, incluindo a comparação dos resultados com os de um estudo anterior realizado por nosso grupo em 2017.

**Métodos** O estudo consistiu em uma análise transversal, envolvendo especialistas em cirurgia de ombro, para investigar o uso de infiltração e bloqueio de nervo no tratamento da dor no ombro. Foi conduzida uma pesquisa com questionários estruturados, abordando a prática clínica em relação a esses procedimentos. Os dados foram coletados e analisados por meio de estatísticas descritivas e análises de associação entre variáveis, como idade dos pacientes e tipo de procedimento realizado.

**Resultados** Os resultados revelaram uma alta taxa de adoção de infiltrações e bloqueios de nervo para o tratamento da dor no ombro, com predomínio de pacientes acima de 40 anos de idade. O uso de corticoides e ácido hialurônico foi comum nas infiltrações, especialmente nos casos de artrose do ombro e lesões parciais do manguito rotador. No entanto, a ultrassonografia não foi amplamente utilizada como guia durante os procedimentos. O bloqueio de nervo, principalmente do nervo supraescapular, foi realizado por dois terços dos entrevistados, com baixa incidência de complicações.

**Conclusão** Este estudo destacou a prevalência e as tendências na prática clínica em relação ao uso de infiltração e bloqueio de nervo no tratamento da dor no ombro. Apesar das lacunas identificadas, como a baixa utilização de ultrassonografia, os resultados fornecem insights valiosos para aprimorar as abordagens terapêuticas e considerar a adoção de tecnologias de imagem na área.

## Introdução

A queixa de dor no ombro frequentemente acomete pacientes adultos, cuja prevalência compreende-se entre 7 e 34%, sendo a população acima de 40 anos a de maior risco. 1 2 Estima-se que de 10 a 16% da população apresente mais de um episódio de ombro doloroso ao longo da vida. 1 3 Questiona-se na prática médica o tratamento, acompanhamento e a recuperação deste tipo de queixa, constando-se que fatores como idade entre 45 e 54 anos e dor por mais de 3 meses inferem mau prognóstico. 1 4 A refratariedade e a recidiva da queixa após 1 ano são complicações presentes em cerca de 40 a 50% dos pacientes. 4 Ao estudar-se a fisiopatologia de acometimentos relacionados ao ombro doloroso, citam-se em primeira importância doenças degenerativas osteotendíneas e alterações inflamatórias como tendinites relacionadas ou não à calcificação. 5 Vale ressaltar que condições como o estágio de degeneração e gravidade das comorbidades possuem relação direta com o prognóstico e desfecho. 5 6

A infiltração e o bloqueio de nervo são procedimentos que mostraram-se efetivos nos últimos anos para controle dos sintomas de dor no ombro, em que os fármacos selecionados são diversos e integram o repertório do médico cuja prática inclui manejo deste tipo de queixa. 7 8 9 Os locais de infiltração, auxílio ou não de métodos de imagem e indicações também são variáveis. 9 10 O objetivo do presente estudo foi avaliar como especialistas de ombro têm utilizado a infiltração e o bloqueio de nervo na sua prática diária.

## Materiais e Métodos

Estudo transversal, caracterizado como uma pesquisa de levantamento interseccional em tempo único, que adotou uma amostragem não probabilística do tipo conveniência. Aprovado pelo Comitê de Ética da nossa instituição sob o número: 71476223.3.0000.5505.

Foi utilizado um questionário, desenvolvido previamente pelos autores deste estudo ( Supplementary Anexo 1 ), que foi administrado durante o 8° Closed Meeting da Sociedade Brasileira de Cirurgia do Ombro e Cotovelo, realizado entre os dias 17 e 19 de agosto de 2023.

Houve a participação de 134 ortopedistas especializados em cirurgia de ombro e cotovelo, de um total de 300 inscritos, resultando em uma taxa de resposta de 44%. Para preservar a confidencialidade, os ortopedistas não foram identificados, sendo-lhes solicitado responder ao questionário apenas uma vez. A distribuição dos questionários ocorreu durante os intervalos dos três dias do congresso, e foi oferecida a possibilidade de resposta tanto de forma virtual, através do Google Forms, quanto de forma física.

As características avaliadas no questionário foram descritas utilizando frequências absolutas e relativas. As condutas de interesse foram analisadas em relação à experiência dos profissionais, com a associação verificada por meio de testes da razão de verossimilhanças. O uso de corticoide em diferentes tipos de procedimentos foi relacionado ao uso de ácido hialurônico em infiltrações, sendo a associação avaliada por meio de testes exatos de Fisher.

As perguntas sobre a experiência dos profissionais e o número de infiltrações realizadas foram descritas em cada uma das pesquisas, e a associação foi avaliada através de testes do qui-quadrado, conforme preconizado por Kirkwood e Sterne (2006).

Para a análise estatística dos dados, utilizou-se o software IBM SPSS Statistics for Windows (IBM Corp., Armonk, NY, EUA), versão 22.0, enquanto a tabulação foi realizada no software Microsoft Excel 2013 (Microsoft Corp., Redmond, WA, EUA). Todos os testes foram conduzidos com um nível de significância de 5%.

## Resultados

Dentre os entrevistados, 29,9% relataram >10 anos de experiência em cirurgia do ombro; 20,1% entre 5 e 10 anos; 37,3% entre 1 e 5 anos; e 12,7%, são cirurgiões com < 1 ano de experiência. Entre a população do estudo, 48,5% relataram de 10 a 30 indicações de infiltração no ano anterior.

As indicações para utilização de infiltração com corticoide subacromial e glenoumeral representam 92,5% e 80,6% do total de indicações de infiltrações, respectivamente. Quanto ao portal de realização da infiltração subacromial, 50,7% optam pelo acesso lateral, 26,9% pelo posterior, e 3% pelo anterior; 18,4% citaram outros acessos. Para a infiltração glenoumeral, 53,7% utilizam ponto de acesso anterior, 29,1% utilizam o posterior, e 17,2% outros acessos.

A infiltração guiada por ultrassonografia não é utilizada para auxílio no procedimento de infiltração pela maioria dos entrevistados (73,1%); 75,4% realizam a infiltração subacromial no consultório. Quanto à infiltração glenoumeral, 45,5% a fazem no consultório, enquanto 19,4% a fazem no centro cirúrgico.

Quando indagados sobre o uso do ácido hialurônico para as infiltrações, 86,6% dos entrevistados utilizam a medicação, sendo que 22% a utilizam para o tratamento de artrose do ombro de forma isolada, e 18,7% para tratamento de lesões parciais do manguito rotador/tendinopatias. A principal complicação encontrada pelos entrevistados foi dor após a infiltração, com 50% na região subacromial e 44,8% na articular.

Em relação ao bloqueio de nervo do ombro, 66,4% dos entrevistados realizam o procedimento, enquanto 33,6% não o realizam; sendo que, 44,8% bloqueiam o nervo supraescapular e 29,9% acrescentam o nervo axilar no bloqueio. Dentre os participantes que realizam bloqueio, apenas 32,1% utilizam o ultrassom para guiar. A maior parte dos participantes relatou que não houve complicações pós-procedimento (36,6%), e, dentre os que relataram complicações, a principal foi a dor local correspondendo a 24,6%.

A Tabela 1 mostra que o maior número de infiltrações no último ano está associado ao maior tempo de experiência dos profissionais ( *p* < 0,001), os profissionais com menos tempo de experiência utilizam menos corticoide na infiltração subacromial e fazem menos infiltrações com ácido hialurônico ( *p* = 0,004 e *p* = 0,041, respectivamente) e os profissionais com maior experiência realizam estatisticamente menos bloqueio de nervo ( *p* = 0,001).

**Tabela 1:** Descrição das condutas tomadas pelos profissionais segundo experiência e resultado dos testes de associação

Variável	1) Quantos anos de experiência possui na cirurgia de ombro?				Tot al	*p*
	< 1 ano	1–5 anos	5–10 anos	>10 anos		
**2) Quantas infiltrações realizou nos últimos 12 meses?**						**< 0,001**
Nenhuma	4 (23,5)	1 (2)	0 (0)	0 (0)	5 (3,7)	
1–10	9 (52,9)	27 (54)	0 (0)	4 (10)	40 (29,9)	
10–30	3 (17,6)	18 (36)	22 (81,5)	22 (55)	65 (48,5)	
> 40	1 (5,9)	4 (8)	5 (18,5)	14 (35)	24 (17,9)	
**8) Realiza a infiltração guiada por ultrassonografia?**						0,079
Não	9 (52,9)	42 (84)	19 (70,4)	28 (70)	98 (73,1)	
Sim	8 (47,1)	8 (16)	8 (29,6)	12 (30)	36 (26,9)	
**12) Qual ou quais medicamentos você utiliza na infiltração subacromial ?**						**0,004**
Sem corticoide	5 (29,4)	3 (6)	2 (7,4)	0 (0)	10 (7,5)	
Com corticoide	12 (70,6)	47 (94)	25 (92,6)	40 (100)	124 (92,5)	
**13) Qual ou quais medicamentos você utiliza na infiltração glenoumeral ?**						0,169
Sem corticoide	4 (23,5)	14 (28)	3 (11,1)	5 (12,5)	26 (19,4)	
Com corticoide	13 (76,5)	36 (72)	24 (88,9)	35 (87,5)	108 (80,6)	
**14) Qual ou quais medicamentos você utiliza na infiltração acromioclavicular ?**						0,542
Sem corticoide	2 (12,5)	2 (4)	1 (3,7)	1 (2,5)	6 (4,5)	
Com corticoide	14 (87,5)	48 (96)	26 (96,3)	39 (97,5)	127 (95,5)	
**16) Realiza infiltração de ácido hialurônico?**						**0,041**
Não	6 (35,3)	6 (12)	1 (3,7)	5 (12,5)	18 (13,4)	
Sim	11 (64,7)	44 (88)	26 (96,3)	35 (87,5)	116 (86,6)	
**20) Realiza bloqueio de nervo?**						**0,001**
Não	4 (23,5)	16 (32)	3 (11,1)	22 (55)	45 (33,6)	
Sim	13 (76,5)	34 (68)	24 (88,9)	18 (45)	89 (66,4)	
**26) Realiza bloqueio do nervo do ombro guiado por ultrassonografia?**						0,666
Não	10 (62,5)	32 (65,3)	16 (64)	11 (50)	69 (61,6)	
Sim	6 (37,5)	17 (34,7)	9 (36)	11 (50)	43 (38,4)	

**Nota:** Teste da razão de verossimilhanças.

A Tabela 2 mostra que não houve associação estatisticamente significativa do uso de corticoide nos procedimentos abordados e a realização de infiltração com uso de ácido hialurônico pelos profissionais ( *p* > 0,05).

**Tabela 2:** Descrição da realização de infiltração com ácido hialurônico segundo o uso de corticoide em cada tipo de procedimento e resultado dos testes de associação

Variável	16) Realiza Infiltração de ácido hialurônico?		Total	*p*
	Não	Sim		
**12) Qual ou quais medicamentos você utiliza na infiltração subacromial ?**				0,133
Sem corticoide	3 (16,7)	7 (6)	10 (7,5)	
Com corticoide	15 (83,3)	109 (94)	124 (92,5)	
**13) Qual ou quais medicamentos você utiliza na infiltração glenoumeral ?**				> 0,999
Sem corticoide	3 (16,7)	23 (19,8)	26 (19,4)	
Com corticoide	15 (83,3)	93 (80,2)	108 (80,6)	
**14) Qual ou quais medicamentos você utiliza na infiltração acromioclavicular ?**				0,590
Sem corticoide	1 (5,6)	5 (4,3)	6 (4,5)	
Com corticoide	17 (94,4)	110 (95,7)	127 (95,5)	

**Nota:** Teste exato de Fisher.

Pela Tabela 3 , tem-se que os profissionais da pesquisa atual têm estatisticamente menos tempo de experiência que os da pesquisa anterior ( *p* = 0,001), e o número de infiltrações realizadas nos últimos 12 meses pelos profissionais da pesquisa atual foi estatisticamente maior que na pesquisa anterior ( *p* < 0,001).

**Tabela 3:** Descrição da experiência e do número de infiltrações nos últimos 12 meses nas duas pesquisas e resultado dos testes de associação

Variável	Pesquisa anterior	Pesquisa atual	*p*
**1) Quantos anos de experiência possui na cirurgia de ombro?**			**0,001**
< 1 ano	17 (9,5)	17 (12,7)	
1–5 anos	35 (19,6)	50 (37,3)	
5–10 anos	43 (24)	27 (20,1)	
> 10 anos	84 (46,9)	40 (29,9)	
**2) Quantas Infiltrações realizou nos últimos 12 meses?**			**< 0,001**
≤ 10	113 (63,1)	45 (33,6)	
> 10	66 (36,9)	89 (66,4)	

**Nota:** Teste do qui-quadrado.

## Discussão

O presente estudo investiga a abordagem de especialistas em cirurgia do ombro no tratamento da queixa de dor nesta região, uma condição prevalente que tem impacto significativo na qualidade de vida dos pacientes. 11 12 O objetivo da pesquisa foi analisar como ortopedistas especializados têm empregado a infiltração e o bloqueio de nervo em sua prática diária, oferecendo uma visão abrangente das tendências e práticas na comunidade médica atual e de forma comparativa do ano de 2020 até o presente em relação ao estudo prévio realizado por nosso grupo, que conta com indagações comuns nos questionários aplicados.

Os resultados revelam uma amostra diversificada de ortopedistas, abrangendo diferentes níveis de experiência, o que se mostra crucial para compreender as variações nas práticas clínicas. A predominância de pacientes acima de 40 anos, conforme identificado na pesquisa, é congruente com a literatura, destacando a relevância dessa condição em uma população mais idosa. 13 14

A pesquisa destaca a prevalência do uso de corticoides em infiltrações subacromiais e glenoumerais, indicando uma prática alinhada com a literatura atual. 15 As preferências quanto ao acesso lateral na infiltração subacromial e o ponto de acesso anterior na infiltração glenoumeral refletem a diversidade nas técnicas utilizadas pelos especialistas. É notável a incorporação do ácido hialurônico em infiltrações, com uma significativa porcentagem de profissionais adotando essa abordagem, principalmente para o tratamento de artrose do ombro e lesões parciais do manguito rotador/tendinopatias. 16 17 Esta tendência sugere uma busca por terapias mais abrangentes e conservadoras no tratamento da dor no ombro.

A maioria dos entrevistados não utiliza ultrassonografia como auxílio no procedimento de infiltração, revelando uma possível lacuna na incorporação de tecnologias de imagem na prática clínica. Esta descoberta suscita discussões sobre a eficácia e a necessidade do uso de métodos de imagem para aprimorar a precisão dos procedimentos. 18 19 Contudo, observou-se o significativo aumento de 15% na utilização da ultrasasonografia como exame auxiliar nas infiltrações de ombro em relação ao estudo anterior realizado em 2020 por nosso grupo.

O bloqueio de nervo do ombro é uma prática comum entre os especialistas, sendo o nervo supraescapular o mais frequentemente bloqueado. 20 A preferência por não utilizar ultrassonografia para guiar o procedimento pode indicar confiança na técnica convencional ou a necessidade de maior conscientização sobre os benefícios da ultrassonografia nesse contexto. 21 A taxa de complicações relatadas é relativamente baixa, com a dor local sendo a complicação mais comum. Esse resultado sugere uma abordagem segura na prática desses procedimentos, mas a análise detalhada das complicações pode fornecer insights valiosos para aprimorar as técnicas e reduzir riscos. 22 23

A associação entre o tempo de experiência e o número de infiltrações realizadas é intrigante. Neste estudo, houve maior número de especialistas com menor tempo de conclusão de formação em relação ao estudo prévio. Os profissionais mais experientes realizam estatisticamente menos bloqueios de nervo, indicando uma possível preferência por outras abordagens terapêuticas ou uma maior efetividade nas decisões de tratamento. É importante destacar que a taxa de resposta de 44% pode introduzir um viés de seleção na amostra, limitando a generalização dos resultados. Ademais, a abordagem convencional em congressos pode influenciar nas respostas dos participantes, uma vez que estes podem estar mais propensos a discutir práticas mais tradicionais.

Comparando este estudo com um estudo anterior conduzido em 2017 durante o 5 ^o^ Closed Meeting da Sociedade Brasileira de Ombro e Cotovelo, quando um questionário foi respondido por 179 ortopedistas especialistas em ombro e cotovelo, observamos diversas similaridades e progressos. 24

Ambos os estudos ressaltam a ampla adoção da infiltração e do bloqueio de nervo como terapias eficazes para mitigar os sintomas da dor no ombro. Entretanto, o presente estudo expande essa análise ao abordar a relação entre a experiência dos profissionais e suas práticas clínicas. Além disso, ambos os estudos destacam a subutilização da ultrassonografia como ferramenta auxiliar nos procedimentos de infiltração, indicando uma possível falha na integração de tecnologias de imagem na rotina clínica.

Ambos os estudos concluem que tanto a infiltração quanto o bloqueio de nervo do ombro são procedimentos seguros, com uma baixa incidência de complicações relatadas. Contudo, este estudo mais recente acrescenta uma nova perspectiva ao explorar a incorporação do ácido hialurônico em infiltrações, sugerindo uma tendência em direção a terapias mais abrangentes e conservadoras. Além disso, adiciona a proloterapia e a aspiração de medula óssea concentrada (BMA, na sigla em inglês) como opções de tratamento, representando terapias inovadoras que ainda estão em avaliação quanto a sua verdadeira relevância e eficácia no tratamento da dor no ombro.

## Conclusão

A prevalência do uso de corticoides e ácido hialurônico em infiltrações indicou uma tendência para abordagens inovadoras e conservadoras. A subutilização da ultrassonografia ressaltou a possível lacuna na adoção de tecnologias de imagem, levantando a necessidade de avaliar a eficácia desses métodos. O bloqueio de nervo do ombro, principalmente do supraescapular, mostrou-se comum e seguro, apesar da baixa incidência de complicações. A associação entre experiência e número de infiltrações sugeriu uma evolução na abordagem ao longo da carreira, com profissionais mais experientes optando por menos bloqueios de nervo. Apesar de limitações, como a taxa de resposta de 44%, o estudo fornece insights valiosos para aprimorar as práticas clínicas na abordagem da dor no ombro por ortopedistas.
